# Supramolecular Iron Complex Formed Between Nitrogen Riched Phenanthroline Derivative and Iron With Improved Oxygen Reduction Activity in Alkaline Electrolyte

**DOI:** 10.3389/fchem.2019.00622

**Published:** 2019-09-13

**Authors:** Lin Gu, Ya Chu, Hongmei Du, Yan Zhang, Jinsheng Zhao, Yu Xie

**Affiliations:** ^1^Shandong Key Laboratory of Chemical Energy Storage and Novel Cell Technology, Liaocheng University, Liaocheng, China; ^2^Key Laboratory of Jiangxi Province for Persistant Pollutants Control and Resources Recycle, Nanchang Hangkong University, Nanchang, China

**Keywords:** oxygen reduction reaction, phenanthroline derivative ligand, Fe complex, electrocatalyst, alkaline electrolyte

## Abstract

In this work, the synthesis and evaluation of a new type non-noble metal oxygen reduction reaction (ORR) catalyst is reported. The catalyst is a complex containing iron ions and multiple N active sites, which displayed excellent oxygen reduction activity in alkaline medium. 2-(2-(4-(1*H*-imidazo[4,5-f][1,10]phenanthrolin-2-yl)pyridin-2-yl)pyridin-4-yl)-1*H*-imidazo[4,5-f][1,10]phenanthroline (PIPhen) was synthesized and used as a ligand to form a rich nitrogen iron coordination complex (Fe-PIPhen), and the complex was then loaded onto the carbon powder to form the target catalyst of Fe-PIPhen/C. The physical characterization of the catalyst was conducted by using Scanning Electron Microscopy (SEM), nitrogen adsorption-desorption and X-ray photoelectron spectroscopy (XPS), Brunauer-Emmett-Teller analysis etc. Electrochemical characterizations were realized by taking cyclic voltammetry (CV), linear sweep voltammetry (LSV) and rotating ring disk electrode (RRDE). The results show that Fe-PIPhen/C possesses the good performance; it exhibits a high electrocatalytic activity, which is mainly via a four electron ORR pathway, with a low hydrogen peroxide yield of 2.58%. And, the average electron transfer number of 3.93 was obtained in alkaline electrolyte. In summary, Fe-PIPhen/C will likely become a promising alternative to Pt catalyst in fuel cell.

## Introduction

As we all know, the pollution caused by the process of energy production and utilization have seriously impair every aspects on human lives. It has become an important international concern to develop new energy technology for energy conservation and environmental protection (Liu et al., 2014, 2016). Among so many kinds of new technologies, proton exchange fuel cells have attracted widely attention in recent years due to the presence of its multiple advantages, such as, high energy conversion efficiency, wide source of fuel and small noise in power generation (Borghei et al., 2018). As the major reaction of fuel cells, oxygen reduction reaction is a extremely significant reaction in the field of electrocatalysis, it is still a challenge for researchers to exploit a highly efficient cathode oxygen reduction reaction catalyst, despite extensive and fruitful research in this field has been undertaken (Lai et al., 2012; Kim et al., 2017). Until now, Pt and its alloys are considered to be the best and most stable catalysts, however, because of its sensitivity to drift over time, high sensitivity to CO (methanol) and high cost (Jafri et al., 2010; Yan et al., 2014), Pt based electrocatalysts are not suitable for large-scale application in fuel cells. Thus, it is necessary to seek out catalysts with high performance, persistent stability and low price to substitute it (Velázquez-Palenzuela et al., 2011; Zheng et al., 2019).

With the emergence, development and application of anion exchange membranes, the advantages of alkaline fuel cells are become increasingly prominent, and the research of alkaline fuel cells is becoming more and more prosperous both in extension and in-depth. Oxygen reduction reaction is an important parts of alkaline fuel cell, and its reaction kinetics is sluggish than that of hydrogen oxidation (Lefevre et al., 2009; Wang et al., 2012; Shen et al., 2018). Therefore, selecting and developing suitable catalyst for cathodic oxygen reduction reaction is the key to improve the performance of alkaline fuel cell and promote the large-scale use of fuel cell (Chen et al., 2018).

Among the non-precious metal catalysts, the transition metal-nitrogen-carbon complex (M-N-C) is the most attractive one to researchers, has been studied in-depth and might be commercially employed in fuel cells in the near future (Artyushkova et al., 2015; Kim et al., 2017; Li J. S. et al., 2017). It has been well accepted that the M-N_x_ unit (M = Fe, Co., etc) is the active sites for the ORR catalytic activity, thus giving the theoretical guidance for fabricating ORR catalysts with more competitive performance (Guo and Xiang, 2017). Compared with the pyrolyzed M-N-C catalyst, the non-pyrolyzed one has advantages in low cost and controllable structures of the as prepared catalysts (Sheelam and Ramanujam, 2018). Many nitrogen ligands have been adopted for the construction of non-pyrolyzed M-N-C catalysts, including macrocycle ligands, such as porphyrins (Banerjee and Nabae, 2019), phthalocyanines (Meng et al., 2019), and metallocorroles (Levy et al., 2015), and also including non-macrocycle ligands, such as phenanthroline (Lu et al., 2015; Ren et al., 2016), bipyridine (Zhao et al., 2018), polypyrrole, polyaniline, etc (Wang et al., 2019). 1,10-phenanthroline (phen) can form the M-N_4_ center with transitional metal ions, and often shows high ORR activity toward the four electron pathway (Ren et al., 2016). However, the complexes are not stable as supported on the carbon black as the ORR catalyst in fuel cells. Immobilization or grafting of phen unit on the supporting materials is of crucial importance to improve the stability of the phen type complex. Electropolymerization method is a facile method for this purpose, where the phen unit can be used as the building block (or side group) of the resultant polymer (He et al., 2017). Besides, the formation of the coordination polymer employing the phenanthroline derivative has also been proved feasible in retaining the active M-N_4_ center in the catalyst (Chu et al., 2018a). A pyrolysis procedure was required for improving the conductivity of coordination polymer, which not only increased the cost, but also led to the significant loss of the M-N_4_ active center (Ma et al., 2019).

At present, the preparation of M-N/C ORR catalyst have achieved some landmark achievements, some of which have comparable properties to that of the Pt based catalysts (Park et al., 2018; Zhang et al., 2018). Nitrogen-containing heterocyclic compounds with thiol groups in side chains, including triazoles and thiadiazole, were deposited on polycrystalline gold electrodes by self-assembly technique and then complexed with copper ions to form ORR catalysts with two-dimensional planar structure. The results show that the activity of the catalysts is closely related to the structure of nitrogen heterocyclic ligands (Kato et al., 2016).

Lin et al. synthesized a bifunctional nitrogen-rich organic ligand, and then further obtained a coordination polymer by complexing the ligand with iron ion. Upon the pyrolysis of the polymer as a precursor, a self-supporting catalyst was obtained with high content of N-binding iron species (Fe-N_x_), which make the catalyst follow the four electron transfer pathway for oxygen reduction with comparable onset and half-wave potentials to that the commercial Pt/C (Lin et al., 2014).

The main objective of the present study is to explore the feasibility of preparing pyrolysis-free coordination polymer as high-efficiency ORR catalyst based on phen unit. For this purpose, we firstly prepared an aromatic nitrogen-rich ligand (PIPhen) with at least three coordination sites, by a simple condensation reaction between 1,10-phenanthroline-5,6-dione and 2,2′-bipyridyl-5,5′-dialdehyde. The full name of the ligand was 2-(2-(4-(1*H*-imidazo[4,5-f][1,10]phenanthrolin-2-yl)pyridin-2-yl)pyridin-4-yl)-1*H*-imidazo[4,5-f][1,10]phenanthroline, which complexed with iron ion in DMF, leading to the formation of the target complex (Fe-PIPhen). Subsequently, the complex was loaded on Vulcan XC-72 by addition of the carbon powder to the above solution under continuous stirring, which enhanced the conductivity and dispersion of the ORR activity.

**Scheme 1:**
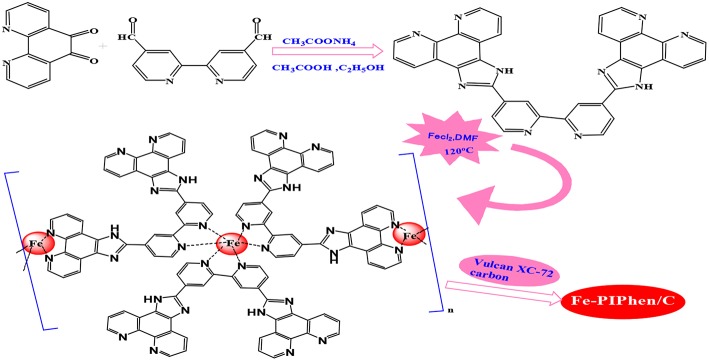
Synthetic route of Fe-PIPhen/C catalyst.

The synthesis diagram of PIPhen and Fe-PIPhen/C was shown in Scheme 1. The pyridinic nitrogen atoms within the phenanthroline and the bipyridine units in the ligand can easily form complexes with transition-metal ions (Such as Cu^2+^, Co^2+^, Fe^2+^). This multi-functional ligand can form coordination polymers by complexing with transition-metal ions, which can improve their stability in some applications, such as ORR catalysis in fuel cells. The high content of the pyridinic nitrogen in the ligand allowed the formation of high content of Fe-N_x_ unit, which is considered to be the active sites for the ORR reactions.

Without the pyrolysis procedure, the as-prepared catalyst Fe-PIPhen/C still exhibit satisfactory ORR activity and high stability, this was witnessed by the four-electron-transfer pathway. This pyrolysis-free process greatly reduces the cost for catalyst production, and endows the Fe-PIPhen/C with potential applications in PEMFCs.

## Experimental

### Materials

1,10-phenanthroline-5,6-dione and 2,2′-bipyridyl-5,5′-dialdehyde were purchased from Zhengzhou Alfachem Co., Ltd. Ammonium acetate, ethanol, glacial acetic acid, N,N-dimethylformamide (DMF), KOH, isopropanol (99.5%), K_3_[Fe(CN)_6_], and FeCl_2_ were purchased from Aladdin Co., Ltd. (Shanghai, China). Carbon (Vulcan XC-72) and Nafion (5 wt% in ethanol) were obtained from Nanjing Hui Yu Energy Technology Co., Ltd. Pure grade N_2_ and O_2_ were implied to received saturated electrode measurement solutions.

### Synthesis of PIPhen and Fe-PIPhen/C Catalyst

In a bottom flask, 0.7925 g (3.77 mmoL) of 1,10-phenanthroline-5,6-dione, 0.40 g (1.88 mmoL) of 2,2'-bipyridyl-5,5'-dialdehyde and 1.5 g (19.4 mmoL) of ammonium acetate were dissolved in 60 mL of ethanol and 10 mL of acetic acid. The solution was refluxed at 120 °C and be stirred by magnetic agitator in argon atmosphere. After 12 h of reaction, the solvent was filtered off, and the filtrate was rinsed with excessive ethanol and deionized water successively, and is dried in a vacuum oven at 60°C, and yellow powder was obtained as the PIPhen ligand.

In order to prepare the Fe-PIPhen/C catalyst, the molar ratio of Fe to PIPhen/C is 9:1, and the specific steps are as follows: 12 mg of PIPhenand 18 mg of ferrous chloride were added into a 100 mL round bottom, and then be dissolved in 50 mL of DMF. Under the argon atmosphere, the solution was kept in the oil bath for 12 h at 120°C. Afterwards, the solution was cooled down to room temperature, and then 100 mg of carbon powder (Vulcan XC-72) was added to the solution and was stirred for 24 h at room temperature. Finally, the solution was poured into 200 mL of distilled water, and placed it for 1 h, and then the solid precipitation was obtained by vacuum filtration. The resulting solid product was dried in the vacuum dryer to obtain our target product, which was named as Fe-PIPhen/C. The synthesis route is exhibited in Scheme 1 (Ma et al., 2018).

### Electrode Preparation and Modification

Before preparing the modified electrode for the catalyst, we firstly need to pre-treat the electrode: the surface of the electrode was polished with Al_2_O_3_ slurry (0.3 μm), and the ultrasonic cleaning was carried out in the ethanol and ultra pure water to achieve the mirror polishing. Secondly, it demands to prepare the Fe-PIPhen/C mixture: 3.2 mg of Fe-PIPhen/C catalyst, 177 μL of isopropanol, 3 μL of nafion, and 570 μL of ultra pure water were added into a 1.5 mL centrifuge tube. The mixture was treated with ultrasonic for 30 min until a uniform ink was formed. Finally, the 8.5 μL of ink was dripped onto a platinum carbon electrode and the electrode was dried at room temperature for subsequent electrochemical tests (Yu et al., 2014).

### Physical Characterization

The surface microscopic characteristics of the catalyst were characterized by the field emission scanning electron microscope (SEM, SU 8020), the accelerating voltage was 3 kV. The sample used for testing was made by dropping the catalyst ink onto to the surface of the Indium Tin Oxide (ITO) coated electrode. The surface element composition of the catalyst was recorded on the Thermo Scientific ESCALAB 250Xi X ray photoelectron spectrometer (XPS), with a monochromatic Al K (1486.6 eV) X ray source, and the catalyst was kept in ultra high vacuum during the measurement (<10^−9^ mbar). After testing, Gauss 4.1 XPS software was used to fit XPS spectra. The N_2_ adsorption-desorption measurements were taken using SI surface area analyzer (Autosorb-Iq-c, United States instrument) at 77 K. The powder X-ray diffraction (PXRD) spectra were measured by a XD-3 Purkinje diffractionmeter, with monochromatized Fe K_a1_ radiation.

### Electrochemical Characterization

The ORR performances of the catalysts are studied by multiple electrochemical method including CV, RDE, and RRDE. The instrument used was AUTOLAB 302 N potentiostat, equipped with AFMSRCE (Pine) rotating disc (ring disk) electrode. The electrochemical tests were conducted by a conventional three electrode and an air flow system with 0.1 M KOH as the test solution (supporting electrolyte). The counter electrode and the reference electrode are Pt ring and Ag/AgCl electrode, respectively. The catalyst modified disc (or ring disk) electrode was used as the working electrode. In advance, the potassium hydroxide solution was aerated for 30 min to obtain oxygen saturation or nitrogen saturation test solution.

## Results and Discussion

### Physical Characterization of the Catalyst

#### IR Test

**Figure 1:**
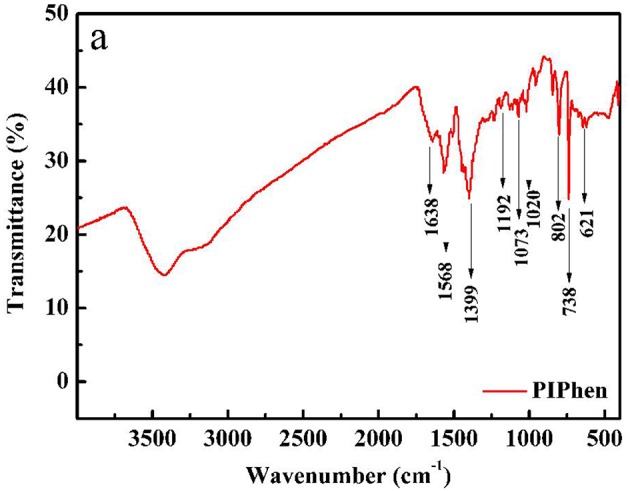
Infrared spectrum of PIPhen.

The ligand was tested by infrared spectroscopy. As shown in Figure 1, the peak at 1638 cm^−1^, 1,568 and 1,399 cm^−1^ belong to the stretching vibration of C = C, C = N bonds or the skeletal vibration of the aromatic rings including the imidazole-phenanthroline ring and the bipyridine ring (Liu et al., 2019). The peaks at 1192, 1073, and 1020 cm^−1^ belong to the in-plane bending vibration of C-H on the aromatic rings. The peaks at 802, 738, 621 cm^−1^ are indication of the out of plane bending vibration of the C-H bonds on the aromatic rings. In summary, we initially judged the successful synthesis of the ligand (Liu et al., 2019). Anal. Calcd for the ligand PIPhen: C, 72.96; H, 3.40; N, 23.64. Found: C, 72.90; H, 3.39; N, 23.71.

#### SEM Measurement

**Figure 2:**
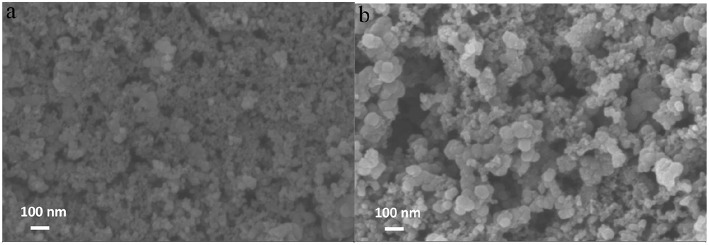
SEM images of Vulcan XC-72 carbon **(A)**, Fe-PIPhen/C **(B)**.

For the sake of acquaintance the surface morphology of the catalyst, SEM was conducted and shown in Figure 2. As the supporting material, the Vulcan XC-72 carbon has an appearance of spherical particles with an average particle size of 35–60 nm (Figure 2A), and the irregular aggregation of the particles forms a porous structure on the surface of the electrode. For Fe-PIPhen/C (Figure 2B) composite, the average radius (40–60 nm) is higher than that of the average radius of Vulcan XC-72 carbon particles, which was the result from the coating of the coordination polymer on the carbon material. In addition, the porous properties of the catalyst can provide more channels for the diffusion of O_2_, H_2_O, H_2_O_2_ and other intermediate products between the surfaces of the electrode, thereby further enhancing the kinetic velocity of electrons and materials (Gu et al., 2017).

#### Brunauer-Emmett-Teller (BET) Measurement of the Catalyst

**Figure 3:**
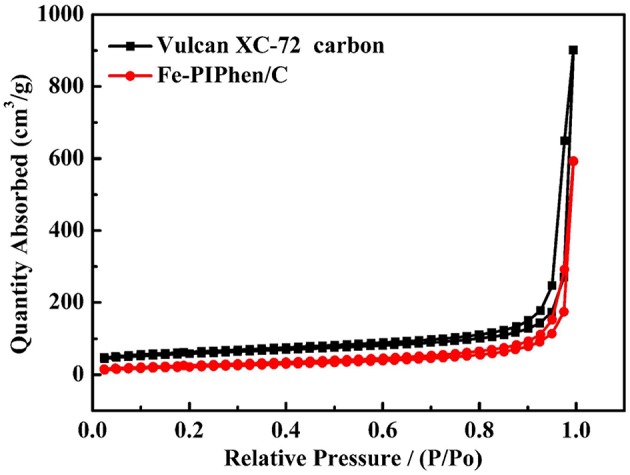
The isotherms of Vulcan XC-72 (black line), Fe-PIPhen/C (red line) during the adsorption/desorption of nitrogen gas.

The porous features of Fe-PIPhen/C were further characterized by N_2_ adsorption-desorption measurements. The N_2_ adsorption-desorption isotherm of Fe-PIPhen/C is shown in Figure 3, it represents the type-III of N_2_ adsorption/desorption isotherms with large specific surface areas and pore volumes (Fan et al., 2017). As summarized in Table 1, the BET surface area and the pore volume of the Vulcan XC-72 carbon and Fe-PIPhen/C are 108.665 m^2^/g, 1.338 cm^3^/g, and 77.849 m^2^/g, 0.90 cm^3^/g, respectively. The two parameters of Fe-PIPhen/C decrease compared with that of Vulcan XC-72 carbon, which represents the successful loading of Fe-PIPhen/C (Yang et al., 2013).

**Table 1:** The parameters obtained from the nitrogen adsorption/desorption of composite.

**Sample property**	**Vulcan XC-72 carbon**	**Fe-PIPhen/C**
BET surface Area (m^2^/g)	108.665	77.849
Pore volume (cm^3^/g)	1.338	0.90

#### XRD Analysis

**Figure 4:**
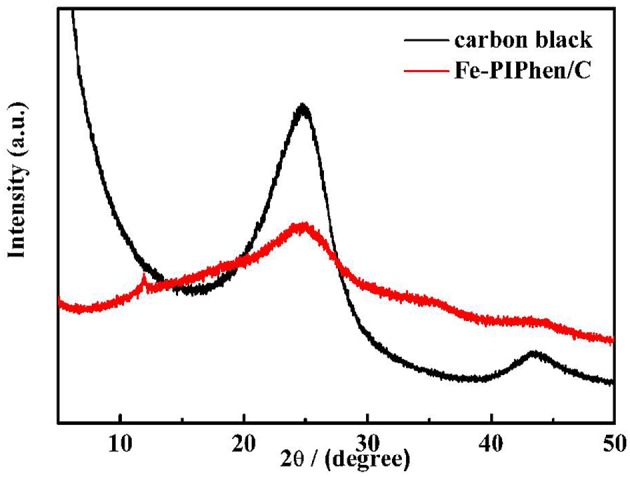
PXRD test curves of Fe-PIPhen/C and carbon black (Vulcan XC-72).

In order to analyze the phase of the prepared material, the powder X-ray diffraction analysis was carried out, as shown in Figure 4, which shows the PXRD test of Fe-PIPhen/C and carbon black (Vulcan XC-72). As can be seen from the Figure 4, the XRD curve of pure carbon has two distinct peaks at 24.8° (002) and 43.6° (110), respectively. After loading the coordination polymer onto the carbon powder, the peak intensity is reduced, but the position of the two broad peaks are almost the same as that of pure carbon, indicating that the loading of the prepared material does not change the crystal structure of carbon black (Vulcan XC-72) to some extent. Coordination polymer is in amorphous state, and its coating on the surface of carbon powder increases the amorphousity of composite, so it causes the decrease in peak intensity and the increase in peak width (Lin et al., 2014). Besides, a small part of coordination polymer orderly agglomerated to give the formation of microcrystal, which was the origin of the diffraction peak at about 11.9°, and also confirmed the formation of the coordination polymer between the Fe^2+^ and the PIPhen ligand.

#### XPS Measurement of the Catalyst

**Figure 5:**
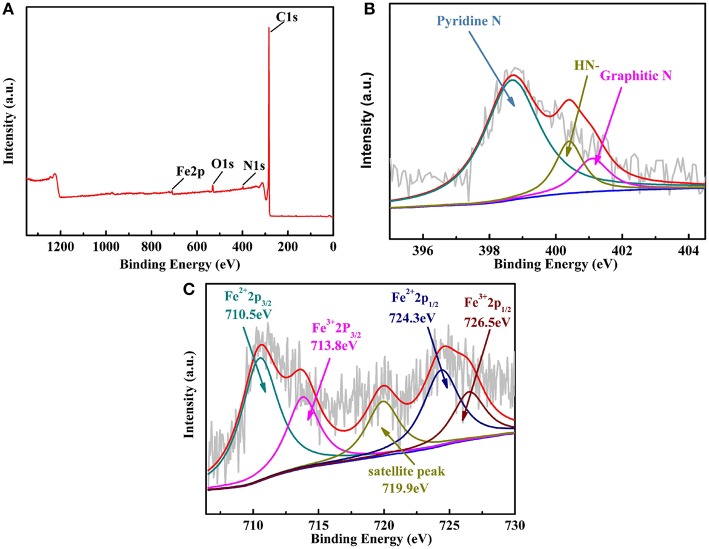
The XPS survey scans of Fe-PIPhen/C **(A)**, the XPS spectrum of N 1s **(B)** and Fe 2p **(C)** for the Fe-PIPhen/C composite.

In order to get the information the elemental compositions and their valence states of Fe-PIPhen/C composite, XPS analysis was carried out, and the result was shown in Figure 5. As shown in Figure 5A, the XPS survey scan results showed the atomic abundance of C, O, N and Fe in the catalyst Fe-PIPhen/C, which are 95.27, 2.39, 1.58, and 0.748%, respectively. The detection of nitrogen and iron atoms indicated the successful loading of the coordination polymers on the carbon powder to some extent (Yang et al., 2011; Guo and Xiang, 2017).

The high resolution of the N1s XPS spectrum (Figure 5B) can be divided into three characteristic peaks (Chen et al., 2017), which are located at 398.6 eV (pyridinic nitrogen), 400.2 eV(= *N*- structure) and 401.1 eV(graphitic N), respectively. Each ligand contains up to eight pyridine nitrogen atoms, which are electron-deficient and each contains a pair of lone pair electrons, thus enabling them to coordinate with transition metal ions. In this case, pyridinic nitrogen is considered to be the complexation sites of the iron ions, forming the Fe-N_x_ unit in the composite catalyst, which has been considered as the active sites for reduction of the oxygen to water in the four electron pathway. In addition, the presence of the N 1s signal at 400.2 eV suggested the presence of the = *N*H- structure in the composite, which is attributed to the = *N*H- structure in the imidazole ring formed from the condensation reaction (Nagase et al., 2019). And, the graphitic N (401.1 eV) might originate from the supporting carbon material (Vulcan XC-72) (Li et al., 2012). As shown in Figure 5C, Fe 2p XPS spectra exhibit multiple peaks, indicating that there are many chemical Fe species in Fe-PIPhen/C catalyst (Wang et al., 2018). The Fe 2p_3/2_ peak at 710.5 eV and the Fe 2p_1/2_ peaks at 724.3 eV suggested the existence of Fe^2+^ ion in the composite (Lu et al., 2015). Accordingly, the Fe 2p_3/2_ peak at 713.8 eV and the Fe 2p_1/2_ peaks at 726.5 eV indicated the presence of the Fe^3+^, which might originate from the oxidation of the Fe^2+^ during the pyrolysis process (Lin et al., 2016). The coexistence of Fe^2+^ ions and pyridinic nitrogen in the coordination polymer in the form of Fe-N_x_ is considered to be the active center of the catalyst for ORR activity. The peak at 719.9 eV is a satellite peak (Lin et al., 2014). Based on the above XPS analysis, it is believed that Fe-PIPhen/C has been successfully synthesized.

### Electrochemical Measurements and Kinetic Study of Catalysts

**Figure 6:**
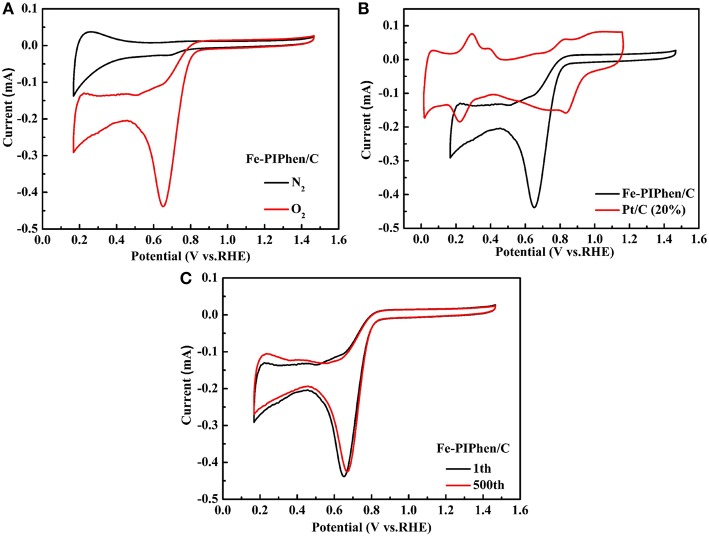
The CV curves of Fe-PIPhen/C **(A)** in nitrogen and oxygen saturated KOH (0.1 M) solution, CV of Fe-PIPhen/C and Pt/C (20% content) in KOH saturated and O_2_ **(B)**, repeated CV curves of Fe-PIPhen/C **(C)** measured in oxygen saturated electrolyte for stability tests. Scan rate: 100 mV/s.

Based on the above physical characterization, we further explored the electrochemical characterization of the catalyst. Firstly, the CV test was carried out in both of the oxygen saturated and nitrogen saturated electrolyte. The potential range of CV was set between −0.8 V and 0.2 V (vs. RHE), and the scanning rate was 100 mV/s (Lefevre et al., 2009). As shown in Figure 6A, the CV curves exhibit the electrochemical activities of Fe-PIPhen/C catalysts in 0.1 M KOH solution saturated with nitrogen or oxygen gas. By comparison, a significantly enhanced reduction peak appears for the CV curve conducted in the oxygen saturated electrolyte, and no apparent reduction peak was observed in the CV of the nitrogen saturated electrolyte. A sharp high reduction peak is observed at +0.65 V (E_p_), and the corresponding current is 0.43 mA. By the analysis and measurements above, it initially signifies that this composite catalyst possesses good catalytic performance for ORR in alkaline solution (Li H. et al., 2017).

In order to further objectively evaluate the electrochemical performance of the catalyst, the catalyst was compared with Pt/C (20%) in the CV measurement (Figure 6B). By comparison, the onset reduction voltage of composite catalyst is 0.79 V, which is somewhat lower than that of Pt/C (0.93 V). On the other hand, the maximum reduction current of the composite catalyst is greater than that of Pt/C (20%), which is approximately three times of the Pt/C catalyst. Therefore, we can find that although the potential of Fe-PIPhen/C is not as good as that of Pt/C (20%), its current has its own advantage.

Furthermore, the stability of the catalyst was evaluated by CV measurement. It was considered that the persistence of the CV curve after repeated cycling directly reflected the stability of the catalyst. As shown in Figure 6C, the reduction current density is approximately decreased by 2.3% after 500 cycles, thus a conclusion could be reached that the Fe-PIPhen/C catalyst has good electrochemical stability (Sivanantham and Shanmugam, 2018).

The electron transfer number involved in ORR reaction was measured by rotating disk electrode (RDE), based on which the mechanism of oxygen reduction was further understood.

**Figure 7:**
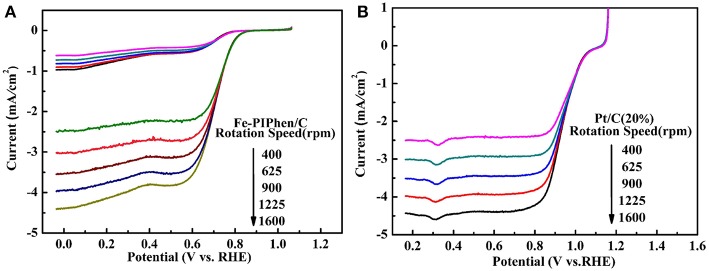
Polarization curves of Fe-PIPhen/C **(A)** and Pt/C (20% content) **(B)** at various rotating rate in KOH solution saturated with O_2_ and N_2_. Scan rate: 10 mV/s.

As depicted in Figures 7A,B, the linear sweep voltammetry (LSV) curves of Fe-PIPhen/C and Pt/C (20% content) are tested at different speeds by RDE measurement (Shen et al., 2017). When the N_2_ saturated electrolyte solution is employed as the electrolyte, the LSV curves correspond to the upper part shown in the Figure 7A, a small reduction current was observed, which may be related to the reduction process of some oxidizing species within the catalyst. On the other hand, an immediate and significant increase in reduction current can be observed upon the nitrogen gas was replaced by O_2_ gas for saturating the electrolyte solution, and the current increases with the increase of rotational speed, the phenomenon was consistent with some of the well-established ORR catalysts reported previously (Yang et al., 2013; Gao et al., 2016).

The positive correlation between the rotational speed and reduction current is related to the increase of the oxygen availability as the rotational speed is increased. The thickness of the diffusion layer decreases, as the increase of the rotational speed, and at the same time the amount of oxygen reaching the electrode in unit time increases, which leads to the increase in the reduction current.

In addition, it can be seen from the diagram that all the polarization curves have two distinct potential regions, that is a mixed dynamic/diffusion controlled area between 0.6 and 0.8 V and a distinct diffusion controlled region (<0.6 V) (Tanaka et al., 2018). Moreover, for the Fe-PIPhen/C catalyst, the ORR onset potential (*E*_onset_) and the limiting diffusion current density (I_ldc_) are 0.85 V and 4.4 mA/cm^2^, respectively. From the LSV curve, we can observe that it has obvious catalytic activity. In order to further compare its catalytic performance, as shown in Figure 7B, the performance of the Pt/C (20% content) was evaluated by the RDE test, from which the *E*_onset_ and the I_ldc_ of the Pt/C (20% content) are 1.06 V and 4.43 mA/cm^2^, respectively. Although the performance of Fe-PIPhen/C catalyst is slightly worse than that of the Pt/C, it will still have certain technological and economic advantages due to its low cost and simple preparation process (Song et al., 2017).

In general, two oxygen reduction pathways could be identified, one is the four electron transfer processes with H_2_O as the destination of oxygen, the other is two electron transfer processes, in which oxygen was transformed to H_2_O_2_. For the two electron pathway, its oxygen utilization efficiency is low, and also the H_2_O_2_ produced has a detrimental effect to the proton exchange membrane due to its oxidative property.

Furthermore, the electron transfer numbers at the different potentials are calculated by the K-L equation as follows (Zhu et al., 2017):

(1) $$\begin{array}{l} 1/i=1/i_{k}+1/{B\omega }^{1/2} \end{array}$$

(2) $$\begin{array}{l} B=0.62nFC_{O_{2}}D_{O_{2}}^{2}/3v^{-1/6} \end{array}$$

where i is the measured current density and i_k_ is the kinetic current density, respectively, ω is the rotating speed, *n* is the electron transfer number, *F* is the Faraday constant (96485 C·mol^−1^), C_O2_ is the bulk concentration of O_2_ (1.2 × 10^−3^ mol·L^−1^), D_O2_ is the diffusion coefficient of O_2_ (1.9 × 10^−5^ cm^2^·s^−1^), and υ is the kinematic viscosity of the electrolyte (0.01 cm^2^·s^−1^).

**Figure 8:**
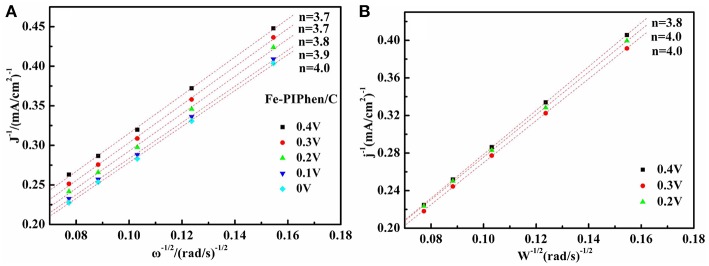
the K-L plots at the potential 0.4 V, 0.3 V, 0.2 V, 0.1V,0 V for Fe-PIPhen/C **(A)**, and for Pt/C (20% content) **(B)** at the potential 0.2 V, 0.3 V and 0.4 V respectively.

Based on the Koutecky-Levich (K-L) equations and the K-L curve (Figure 8A), the average transfer electron number (n) in the process is calculated to be 3.7, 3.7, 3.8, 3.9, 4.0 at the 0.4 V, 0.3 V, 0.2 V, 0.1 V, 0 V for Fe-PIPhen/C, respectively, indicating that the ORR catalyzed by Fe-PIPhen/C occurs through the four electron reduction pathway, which closed to the 4.0 of commercial Pt/C (20% content) (Figure 8B) (Ren et al., 2016), it may become a promising candidate to Pt/C (20% content) in the field of fuel cells.

**Figure 9:**
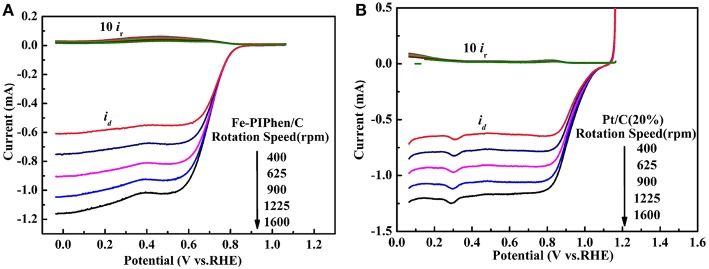
Rotating ring disk electrode (RRDE) curves of Fe-PIPhen/C **(A)**, and Pt/C (20% content) **(B)**. Scan rate: 10 mV/s.

As is known to all, the four electron transfer process is excellent than the two electron transfer process, the reason of which is that hydrogen peroxide has a corrosive effect on fuel cells and the stability of the electrocatalyst layer may be damaged. In order to further confirm the test results of RDE and calculate the yield of H_2_O_2_ in the reaction process, we carried out the RRDE measurements. The range of the disk voltage is −0.1 V−1.1 V (vs. RHE), and the ring electrode voltage is set to 0.6 V (vs. Ag/AgCl/ saturated KCl) to ensure that the hydrogen peroxide produced on the disk electrode is completely oxidized into water on the ring electrode (Sun et al., 2017). The RRDE curve is shown in Figures 9A,B. The upper part of the diagram is the ring electrode current, and the lower part is the disk electrode current. The ring electrode current increases with the increase of rotational speed, which implied that the amount of H_2_O_2_ detected increased. In order to further confirm the total number of electrons catalyzed by ORR and the percentage of H_2_O_2_ detected by RRDE in O_2_ reduction process. We can determine the number of electrons (n) and hydrogen peroxide yield (%H_2_O_2_) according to the following equation (Choi et al., 2017).

(3) $$\begin{array}{l} n=\frac{4-\left(Ir/Id\right)}{N} \end{array}$$

(4) $$\begin{array}{l} \text{\%}{\text{H}}_{2}{\text{O}}_{2}=\frac{\left(Ir/Id\right)}{N}\times 100 \end{array}$$

Among them, I_r_ and I_d_ are ring electrode current and disk electrode current (mA), respectively, and N is H_2_O_2_ collecting efficiency on ring electrode (*N* = 0.37) (Sun et al., 2017).

**Figure 10:**
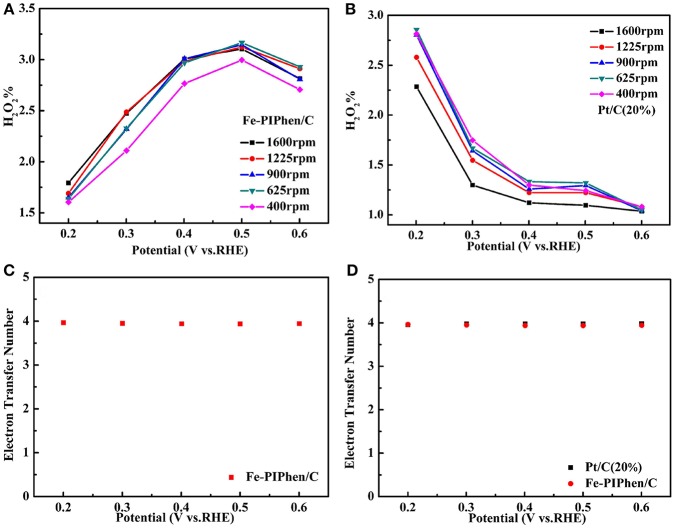
Hydrogen peroxide yield of Fe-PIPhen/C **(A)** and Pt/C(20% content) **(B)** at various rotating rate, respectively, the ETN for Fe-PIPhen/Cat different potentials (1600 rpm) **(C)**, the ETN for Fe-PIPhen/C and Pt/C(20% content) at different potentials (1600 rpm) **(D)**.

As exhibited in Figures 10A,B, the H_2_O_2_ yield of Fe-PIPhen/C and Pt/C (20% content) are about 1.6–3.1 and 1.03–2.81%, respectively, and the voltage differences from 0.2 V to 0.6 V. It can be seen from the comparison that the H_2_O_2_ content of Fe-PIPhen/C is basically equal to that of Pt/C (20% content), indicating that the H_2_O_2_ produced by Fe-PIPhen/C in the process of oxygen reaction reduction is very few, which suggested that it mainly follows the four electron transfer pathway. Furthermore, as shown in Figure 10C, the average number of electron transfer number of Fe-PIPhen/C under five different voltages at the speed of 1600 rpm is 3.94, which is comparable to that of the Pt/C catalyst (Figure 10D), indicating the nearly complete reduction of oxygen into water. The ORR performance of the as-prepared catalysts was also compared with the previous reported ORR catalysts based on organic ligands and transitional metal ions. As shown in Table 2, Fe-PIPhen/C showed a competitive performance among these catalysts.

**Table 2:** The electrocatalytic parameters of non-precious metal catalysts for ORR reported in recent years.

**Catalyst**	**E_**peak**_ (V)**	**E_**onset**_ (V)**	**ETN**	**Average H_**2**_O_**2**_ (%)**	**Electrolyte solution**	**Reference electrode**	**Refs**.
Fe-PIPhen/C	0.65	0.85	3.8	2.35%	0.1 M KOH	RHE	This work
Pt/C (20 %)	0.80	1.06	4.0	1.5%	0.1 M KOH	RHE	This work
Fe/SNC	0.68	0.86	3.9	**<**6%	0.5 M H_2_SO_4_	RHE	[a]
Pt_30_/FeNC	0.68	0.83	–	–	0.1 M HClO_4_	RHE	[b]
AgDS-PANI	0.76	0.85	4.0	–	0.1 M KOH	RHE	[c]
(Fe,Mo)–N/C-3	0.71	0.84	4.12	–	0.5 M H_2_SO_4_	RHE	[d]

*[a] Shen et al. (2017); [b] Park et al. (2018); [c] Zhang et al. (2017); [d] Lin et al. (2016)*.

**Figure 11:**
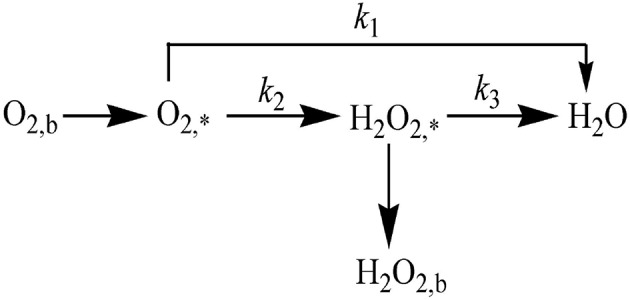
The available routes for ORR. Footnotes b and * refers to the bulk solution substance and electrode surface substance, respectively.

In addition, we can utilize the data obtained by RRDE to further quantify the ORR mechanism. As reported in the literature (Parimi et al., 2011), the proposed reaction pathway of ORR can be elaborately described in Figure 11. The reaction paths involved in each step and the corresponding reaction rates *k*_1_, *k*_2_, and *k*_3_ are exhibited in the below.

According to the literatures (Thomas et al., 2016), the four electron pathway could be realized by two routes: one is the direct four electron pathway without the intermediate product of that is, oxygen is directly converted into water (Equation 5). The other is an indirect pathway, oxygen is firstly converted into H_2_O_2_ by two electron reduction, and then H_2_O_2_ produced is further converted into H_2_O through the two electron way, which is called the “one by one” reaction pathway (Equations 6, 7).

(5) $$\begin{array}{l} {\text{O}}_{2}+4{\text{H}}^{+}+4{\text{e}}^{-}\overset{k_{1}}{\to }2{\text{H}}_{2}\text{O} \end{array}$$

(6) $${\text{O}}_{2}+2{\text{H}}^{+}+2{\text{e}}^{-}\overset{k_{2}}{\to }{\text{H}}_{2}{\text{O}}_{2}$$

(7) $${\text{H}}_{2}{\text{O}}_{2}+2{\text{H}}^{+}+2{\text{e}}^{-}\overset{k_{3}}{\to }2{\text{H}}_{2}\text{O}$$

The rate constants of the catalysts at different potentials: *k*_1_, *k*_2_, and *k*_3_ can be calculated from the following formula (Equations 8–13). The intercept and slope obtained by the relationship of I_d_/I_r_ and ω^−1/2^ are I_1_ and S_1_ values, respectively. In addition, the relationship between I_dl_/(I_dl_-I_d_) and ω^−1/2^ is also plotted and simulated, and the slope acquired is S_2_ (Chu et al., 2018b). I_d_ and I_r_ is the disk limiting diffusion current and the ring disk current, respectively, and I_dl_ can be calculated from the Equations (1, 2).

(8) $$\frac{{\text{I}}_{\text{d}}}{{\text{I}}_{\text{ r}}}=\frac{1+2k_{1}/k_{2}}{\text{N}}+\frac{2\left(1+k_{1}/k_{2}\right)}{{\text{NZ}}_{\text{H}}2{\text{O}}_{2}}k_{3}{\text{ω}}^{-1/2}$$

(9) $$\begin{array}{l} \frac{{\text{I}}_{\text{dl}}}{I_{dl}-I_{d}}=1+\frac{k_{1}+k_{2}}{{\text{Z}}_{{\text{O}}_{2}}}\omega^{-1}/2\;; \end{array}$$

(10) $$\begin{array}{l} k_{1}={\text{S}}_{2}{\text{Z}}_{{\text{O}}_{2}}{\frac{{\text{I}}_{1}\text{N}-1}{\text{I}}}_{1}\text{N}+1 \end{array}$$

(11) $$\begin{array}{l} k_{2}=\frac{2{\text{Z}}_{{\text{O}}_{2}}S_{2}}{I_{1}N+1} \end{array}$$

(12) $$\begin{array}{l} k_{3}=\frac{{\text{Z}}_{{\text{H}}_{2}{\text{O}}_{2}}\text{N}{\text{S}}_{1}}{{\text{I}}_{1}\text{N}+1} \end{array}$$

(13) $$\begin{array}{l} {\text{Z}}_{{\text{H}}_{2}{\text{O}}_{2}}=0.62{\text{D}}_{{\text{H}}_{2}{\text{O}}_{2}}^{2/3}v^{-1/6}{\text{ Z}}_{{\text{O}}_{2}}=0.62{\text{D}}_{{\text{O}}_{2}}^{2/3}v^{-1/6} \end{array}$$

D_H_2_*O*_2__ and D_O2_ are the diffusion coefficients of H_2_O_2_ and O_2_ in the KOH solution, respectively, which were 1.83 × 10^−5^ cm^2^·s^−1^ and 1.9 × 10^−5^ cm^2^·s^−1^, respectively. ν is the kinematic viscosity of 0.1 M KOH solution, which was cited as 0.01 cm^2^s^−1^ (Artyushkova et al., 2015).

**Figure 12:**
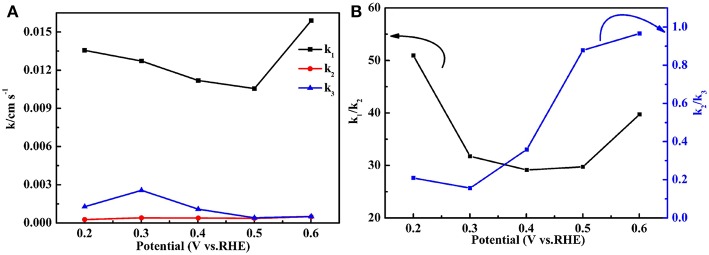
The k_1_, k_2_, and k_3_ values of the Fe-PIPhen/C catalyst **(A)** and the values of k_1_/k_2_ and k_2_/k_3_ **(B)**.

Figure 12A exhibits the values of *k*_1_, *k*_2_, and *k*_3_ of the Fe-PIPhen/C catalyst at different voltages, which showed the order of *k*_1_ >> *k*_2_≈*k*_3_ from the diagram. *k*_1_ is far larger than *k*_2_, and *k*_3_, and *k*_2_ is approximately equal to *k*_3_. These data indicate that most of the oxygen may be directly reduced to H_2_O at a very fast rate, and a small part of the O_2_ is converted to H_2_O_2_ at a much slower rate, which is consistent with a higher ETN value and a lower H_2_O_2_ yield of the Fe-PIPhen/C catalyst. In order to compare their values more intuitively, the ratios of *k*_1_/*k*_2_ and *k*_2_/*k*_3_ are displayed in Figure 12B, which shows that the ranges of *k*_1_/*k*_2_ and *k*_2_/*k*_3_ are 30–50 and 0.2–1.0, respectively, these data further indicate that *k*_1_ >> *k*_2_≈*k*_3_, and supporting the conclusion discussed above. Finally, the results show that the main reaction route of the Fe-PIPhen/C catalyst is the direct four electron transfer process.

## Conclusions

Herein, we designed a low cost and high activity non-noble metal electrocatalyst for oxygen reduction in alkaline electrolyte. A nitrogen rich ligand PIPhen was chosen as a precursor, which was reacted with Fe^2+^ to form the coordination polymer (Fe-PIPhen). The ORR catalyst Fe-PIPhen/C was formed by loading the coordination polymer on the carbon powder, which was free of the pyrolysis process. The electrochemical results show that the prepared Fe-PIPhen/C catalyst possesses high ORR activity and the onset reduction potential is about 0.85 V, slightly lower than that of the Pt/C catalyst. In addition, Fe-PIPhen/C follows a direct four electron reduction pathway, directly converting oxygen molecular to water with a high reaction rate. The work here shows that the Fe-PIPhen/C catalyst is one of the most promising alternatives to the Pt/C catalyst in the fuel cell ORR process, due to its low cost and easy fabrication process.

## Data Availability

The datasets generated for this study are available on request to the corresponding author.

## Author Contributions

YC performed experiments and drafted the manuscript. LG took the instrumental analysis and analyzed the data. HD helped to discuss some of the experimental results. YZ discussed the mechanisms of the photocatalysis reaction. JZ provided the idea and guided the undertaken of the experiments. YX polished the language of the manuscript.

### Conflict of Interest Statement

The authors declare that the research was conducted in the absence of any commercial or financial relationships that could be construed as a potential conflict of interest.
